# The caspase inhibitor zVAD increases lung inflammation in pneumovirus infection in mice

**DOI:** 10.14814/phy2.12332

**Published:** 2015-03-16

**Authors:** Elske van den Berg, Suzanne M Bal, Maria T Kuipers, Gustavo Matute-Bello, René Lutter, Albert P Bos, Job B M van Woensel, Reinout A Bem

**Affiliations:** 1 Pediatric Intensive Care Unit, Emma Children's Hospital, Academic Medical CenterAmsterdam, The Netherlands; 2 Department of Respiratory Medicine and Experimental Immunology, Academic Medical CenterAmsterdam, The Netherlands; 3 Laboratory of Experimental Intensive Care, Academic Medical CenterAmsterdam, The Netherlands; 4 Division of Pulmonary and Critical Care Medicine, the Center for Lung Biology, University of WashingtonSeattle, Washington, USA

**Keywords:** Acute respiratory distress syndrome, apoptosis, neutrophils, respiratory syncytial virus

## Abstract

Severe respiratory syncytial virus (RSV) disease is a frequent cause of acute respiratory distress syndrome (ARDS) in young children, and is associated with marked lung epithelial injury and neutrophilic inflammation. Experimental studies on ARDS have shown that inhibition of apoptosis in the lungs reduces lung epithelial injury. However, the blockade of apoptosis in the lungs may also have deleterious effects by hampering viral clearance, and importantly, by enhancing or prolonging local proinflammatory responses. The aim of this study was to determine the effect of the broad caspase inhibitor Z-VAD(OMe)-FMK (zVAD) on inflammation and lung injury in a mouse pneumovirus model for severe RSV disease. Eight- to 11-week-old female C57BL/6OlaHsd mice were inoculated with the rodent-specific pneumovirus pneumonia virus of mice (PVM) strain J3666 and received multiple injections of zVAD or vehicle (control) during the course of disease, after which they were studied for markers of apoptosis, inflammation, and lung injury on day 7 after infection. PVM-infected mice that received zVAD had a strong increase in neutrophil numbers in the lungs, which was associated with decreased neutrophil apoptosis. Furthermore, zVAD treatment led to higher concentrations of several proinflammatory cytokines in the lungs and more weight loss in PVM-infected mice. In contrast, zVAD did not reduce apoptosis of lung epithelial cells and did not affect the degree of lung injury, permeability, and viral titers in PVM disease. We conclude that zVAD has an adverse effect in severe pneumovirus disease in mice by enhancing the lung proinflammatory response.

## Introduction

The human pneumovirus, respiratory syncytial virus (RSV) is the most common pathogen causing lower respiratory tract disease (LRTD) in infants and young children (Hall et al. 2009). The burden of RSV disease is high; recently it was estimated that annually over 3.0 million young children with RSV-LRTD need to be hospitalized worldwide (Nair et al. 2010). In severe cases, RSV-induced LRTD can lead to respiratory failure and the need for mechanical ventilation. Many of these children fulfill the criteria for acute respiratory distress syndrome (ARDS) (Bachmann and Pfenninger 1994; Dahlem et al. 2003), a life-threatening pulmonary condition that is characterized by hypoxic respiratory failure caused by massive neutrophilic inflammation and alveolar epithelial injury (Ware and Matthay 2000; Ranieri et al. 2012).

Several studies in both humans and animals have shown that severe pneumovirus disease is associated with increased apoptosis, or regulated cell death, of lung airway and alveolar epithelial cells [reviewed by van den Berg et al. (2013)]. Welliver and coworkers found extensive expression of cleaved (active) caspase-3, a marker of classical (caspase-dependent) apoptosis, in epithelial cells in the lungs of children with fatal RSV-LRTD (Reed et al. 2008; Welliver et al. 2008). Likewise, apoptotic features such as increased DNA fragmentation, an apoptotic morphological feature, and caspase-3 activation are detected in the lung epithelium of calves infected with bovine RSV (Viuff et al. 2002) and in mice infected with the rodent-specific pneumovirus pneumonia virus of mice (PVM) (Bem et al. 2010a, 2010c). Extrinsic proapoptotic mediators may be involved in caspase-dependent cell death in pneumovirus infection, including the death receptor ligands TNF-related apoptosis-inducing ligand (TRAIL) (Kotelkin et al. 2003; Bem et al. 2010a) and Fas ligand (FasL) (O'Donnell et al. 1999; Welliver et al. 2008), and the serine proteases granzymes (Bem et al. 2008, 2010c).

As the airway epithelium is the main site of pneumovirus replication, apoptosis leading to enhanced viral clearance may be an important host defense mechanism (van den Berg et al. 2013). An overshoot, however, and/or inefficiency of proapoptotic signaling may contribute to disease. For example, extensive sloughing of dead bronchial epithelial cells, forming dense plugs together with fibrin and mucus, can lead to small airway obstruction (Johnson et al. 2007), a clinical characteristic of RSV-LRTD. In addition, although viral replication mainly occurs in the airways, in the late and severe phase of pneumovirus disease apoptosis is also observed on a wide scale in the alveolar epithelium, suggesting the occurrence of bystander lung epithelial injury (van den Berg et al. 2013), similar to findings in human ARDS and animal models of lung injury (Martin et al. 2005). As such, lung epithelial cells apoptosis may be an important pathogenic event in severe hypoxic RSV-LRTD.

From the above, one can hypothesize that treatment strategies to block apoptosis in the lung may protect the lung epithelium and thus improve the outcome of severe RSV-LRTD. Indeed, experimental studies in animals modeling human ARDS have shown that inhibition of apoptosis in the lungs can be beneficial in terms of survival, lung permeability, and histopathological alterations (Kawasaki et al. 2000; Matute-Bello et al. 2005; Lipke et al. 2010; Messer et al. 2013). The various treatment strategies to inhibit apoptosis used in these studies include silencing of caspases and death receptor signaling by small interfering RNA (Perl et al. 2005; Messer et al. 2013), as well as blockade by decoy receptors and fusion proteins instilled in the lungs (Matute-Bello et al. 2005). Interestingly, systemic administration of the synthetic peptide Z-VAD(OMe)-FMK (zVAD), a widely used irreversible pan-caspase inhibitor, reduced lung epithelial cell apoptosis (Kawasaki et al. 2000; Le Berre et al. 2004; Herrero et al. 2013), lung permeability (Le Berre et al. 2004; Lipke et al. 2010; Herrero et al. 2013), and mortality (Kawasaki et al. 2000) in several animal models of lung injury.

On the other hand, it is important to consider that the use of apoptosis inhibitors in the lungs during RSV-LRTD may also have deleterious effect for the host. First by compromising viral clearance, and second by promoting local inflammatory responses through prolonging the life span of potentially harmful leukocytes. This may be in particularly relevant to neutrophils, which are the most abundant cells in the lungs during RSV-LRTD (McNamara et al. 2003). Apoptosis inhibitors, like zVAD can decrease neutrophil apoptosis in vitro and in vivo (Rossi et al. 2006; D'Avila et al. 2008; Luo and Loison 2008; Wardle et al. 2011). Prolonged or increased neutrophil activation has been implicated as a key pathogenic event in ARDS (Matute-Bello et al. 2000; Martin 2002; Matthay et al. 2012). In addition, apoptosis inhibitors such as zVAD were shown to enhance lung proinflammatory cytokine release in vivo, independent of its antiapoptotic effects (Herrero et al. 2013). As such, apoptosis inhibitors may have strong adverse effects by contributing to lung inflammation.

In the present study, we investigated the effects of zVAD on lung inflammation and lung injury in mice with pneumovirus infection. PVM infection in mice was used to model severe RSV-induced LRTD as previously described (Bem et al. 2011). This cognate host-pneumovirus model has several advantages specifically related to the study question, including high viral replication and marked caspase-3 activation in the lungs (Bem et al. 2010c, 2011). Our hypothesis was that zVAD treatment would reduce lung injury in PVM-infected mice, by protecting against lung epithelial apoptosis.

## Materials and Methods

### Viral stock preparation

The fully pathogenic PVM strain J3666 was originally obtained from Dr. A. J. Easton (University of Warwick, Coventry, UK) from virus stocks originating at the Rockefeller University. Virulence was maintained by continuous passage in mice (Domachowske et al. 2000). The titer of the viral stock used in this work was 12 × 10^4^ copies of PVM per microliter as determined by quantitative real-time PCR (see below). Just prior to experiments, PVM was diluted in Roswell Park Memorial Institute Medium (RPMI) 1640 medium (Invitrogen, Paisley, UK) in a total volume of 80 *μ*L to be delivered by intranasal inoculation.

### Reagents

The broad caspase inhibitor zVAD was obtained from Bachem, Basel, Switzerland. A stock preparation of 20 mg/mL was prepared in sterile DMSO to solubilize the compound. On the day of the experiment, the stock was diluted in PBS to a final DMSO concentration of 5%. For control experiments, a solution containing 5% DMSO in PBS (vehicle), was used. The endotoxin concentration in the dissolved and diluted zVAD was below 0.25 EU/mL (assay lower limit) as determined by a ToxinSensor Gel Clot Endotoxin Assay Kit (GenScript, Piscataway, NJ).

The soluble, human, recombinant SuperFas Ligand (rh-sFasL) was obtained from Enzo Life Sciences (Raamsdonksveer, the Netherlands).

### Animal protocol

All animal protocols were approved by the Institutional Animal Care and Use Committee of the University of Amsterdam, the Netherlands. The mice were maintained under specific pathogen-free conditions according to guidelines of our university. Eight- to 11-week-old female C57Bl/6OlaHsd (C57BL/6) mice (Harlan, Venray, the Netherlands) were used in all experiments.

On day zero, 9.6 × 10^3^ copies of PVM strain J3666 were delivered via intranasal instillation to mice anesthetized with isoflurane. Five-and-a-half days after PVM infection, when first caspase activation is observed, the mice received either 10 mg/kg of the broad caspase inhibitor zVAD or 5% DMSO in PBS (vehicle) subcutaneously in a volume of 10 mL/kg. Administration of zVAD or vehicle was repeated every twelve hours for a total of three doses. The mice were studied on day 7 after PVM infection, 12 h after the last zVAD or vehicle injection. In additional experiments, mice were studied with different zVAD doses ranging from 2 to 10 mg/kg, and with a higher PVM inoculum of 9.6 × 10^4^ copies to obtain more severe PVM disease. In this latter experiment, zVAD treatment in a similar dosing regimen was started at four-and-a-half days and mice were studied on day 6 after PVM inoculation.

Finally, in separate experiments, caspase-3 inhibition by zVAD administration was confirmed in a model of Fas-induced lung injury in mice (Herrero et al. 2013). For this, C57BL/6 mice received 25 ng/g bodyweight of rh-sFasL by intratracheal instillation. Briefly, the mice were anesthetized with inhaled isoflurane and intubated endotracheally with a 22-gauge InsyteTM angiocath (BD, Madrid, Spain) as described before (van den Berg et al. 2011). Six hours before and 6 h after intratracheal instillation of rh-sFasL the mice received either 10 mg/kg zVAD in 5% DMSO or 5% DMSO in PBS (vehicle) subcutaneously. The mice were euthanized 12 h after rh-sFasL instillation. Mice that received no treatment served as controls.

At the end of these experiments, the mice were euthanized with an ip injection containing ketamin, medetomidin, and atropin and exsanguinated by carotid artery ligation. The left lung was removed and flash-frozen in liquid nitrogen for homogenization. The right lung was lavaged with PBS containing 0.6 mmol/L EDTA, one 0.6-mL aliquot, followed by two 0.5-mL aliquots. After the lavage, the lung was fixed in 10% formalin for histological studies. In separate experiments, we obtained single-cell suspensions of the lungs for flow cytometry analysis.

### Measurements

#### Clinical response

Total body weight and a clinical scoring system previously described in multiple PVM mouse studies were used as previously described (Bem et al. 2010c): 1 = healthy, no signs of illness, 2 = subtle ruffled fur, 3 = evident ruffled fur with hunched posture, 4 = evident lethargy with abnormal breathing pattern, 5 = moribund, 6 = death [modified from Cook et al. (1998)]. The end point for sacrifice used in this study was a score of 4 and/or loss of >20% of starting body weight.

#### Lung histology

The degree of lung injury was graded in a blinded fashion on hematoxylin and eosin-stained lung sections, according to the histology scoring system of the American Thoracic Society workshop on experimental lung injury in animal models (Matute-Bello et al. 2011). Briefly, 20 random high-power fields (400 × total magnification) of every lung tissue section were examined for the presence of neutrophils in the alveolar space or in the interstitial space, hyaline membranes, proteinaceous debris filling the airspaces, and alveolar septal thickening. To generate the lung injury score, the sum of each of the five independent variables was weighted according to relevance of each feature and normalized to the number of fields that were counted according to the formula described in (Matute-Bello et al. 2011).

#### Lung inflammation

Total cell counts and differentials in broncho-alveolar lavage fluid (BALF) were performed as described previously (van den Berg et al. 2011). The concentrations of IFN-gamma and KC (CXCL1) were measured with immunoassays (eBioscience, Hadfield, UK and R&D systems, Minneapolis, MN). Monocyte chemotactic protein-1 (MCP-1), IL-6, IL-12, IL-13, G-CSF, macrophage inflammatory protein-1beta (MIP-1 beta) and RANTES were measured by multiplex fluorescent bead assay using the BioPlex Pro Mouse Cytokine 23-plex immunoassay (Bio-Rad Laboratories, Veenendaal, the Netherlands).

#### Lung permeability

The high-molecular-weight protein IgM was measured in BALF by enzyme-linked immunosorbent assay (Holter et al. 1986; Matute-Bello et al. 2011) (Bethyl Laboratories, Montgomery, TX).

#### Lung apoptosis

Quantification of the overall lung caspase-3 activity in lung homogenates and caspase-3 immunohistochemistry on lung sections was performed as described previously (Bem et al. 2010c). To quantify lung epithelial cell apoptosis by immunohistochemistry, the number of cleaved caspase-3-positive epithelial cells (airway and alveolar) was counted in four random high-power fields per lung tissue section.

To obtain single lung cell suspensions for FACS analysis, lungs were perfused with 20 mL PBS through the right ventricle, minced using iridectomy scissors, and digested with collagenase III. Finally, the cells were passed through a 70-*μ*m cell strainer and subjected to RBC lysis, and kept on ice until labeling. The isolated cells from the lung and BALF were Fc-blocked with antimurine CD16/CD32 and labeled with: Annexin V-APC (A35110; Life-Technologies-Invitrogen, Bleiswijk, the Netherlands), LIVE/DEAD fixable far red dead cell stain kit (Life-Technologies-Invitrogen, Bleiswijk and the Netherlands) and the neutrophil marker Ly-G6 (GR1-PE; eBioscience, Hatfield, UK). Annexin V-positive and LIVE/DEAD far red stain-negative cells were considered apoptotic.

#### Lung virus titers

Detection of copies of the PVM SH gene by qPCR was performed as previously described (Bem et al. 2010c). The PVM SH gene (GenBank No. AY573815) was used to detect lung viral titers. RNA was isolated from frozen lungs with the RNeasy Mini Kit (Qiagen, Venlo, the Netherlands) and treated with DNase I (Qiagen, Venlo, the Netherlands). Two micrograms of RNA was reverse transcribed to cDNA using random hexamers (high-capacity cDNA reverse transcription kit; Applied Biosystems, Foster City, CA). Copies of the PVM SH gene were detected in qPCR reactions containing 1 *μ*L of cDNA, Taqman PCR Master Mix (Applied Biosystems), 77 nmol/L TAMRA probe (5′-6FAM-CGCTGATAATGGCCTGCAGCA TAMRA-3′), and 200 nmol/L primers (5′-GCCTGCATCAACACAGTGTGT-3′ and 5′-GCCTGATGTGGCAGTGCTT-3′). The GAPDH housekeeping gene was detected in cDNA samples using rodent GAPDH primers (100 nmol/L) and VIC probe (200 nmol/L) (Ambion, Austin, TX; Applied Biosystems). Standard curves with known concentrations of the full-length SH gene and GAPDH decatemplate (Applied Biosystems) were used for quantification. Results are expressed as copies of PVM SH per 10^9^ copies of GAPDH.

### Statistical analysis

The data were analyzed using GraphPad Prism 4.0 software (San Diego, CA). Comparisons between two groups were performed with the unpaired t-test or the Mann–Whitney U-test depending on data distribution. A *P* value of <0.05 was considered statistically significant. Data are reported as means ± standard error of the mean from three independent replicate experiments with a total of nine mice per group (zVAD or vehicle), unless otherwise specified in the figure legend.

## Results

### Treatment with zVAD increases the total lung neutrophil count in PVM-infected mice, in part by inhibiting neutrophil apoptosis

**Figure 1:**
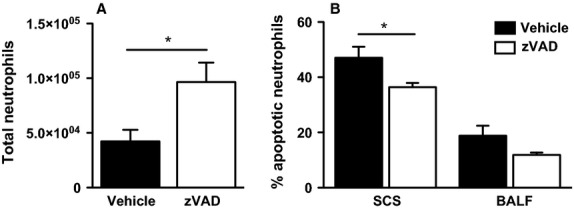
zVAD treatment enhances the neutrophilic response in the lungs during PVM infection. (A) Total BALF neutrophils of vehicle or zVAD-treated mice on day 7 after PVM inoculation. *P < 0.05. Data are shown as means ± SE of three independent replicate experiments of three mice/group each, for a total of 9 mice/group. (B) Percentage of apoptotic neutrophils, as analyzed using flow cytometry, in lung single-cell suspensions (SCS) and BALF of vehicle (black bars) or zVAD (white bars)-treated mice on day 7 after PVM inoculation. *P < 0.05. Data are shown as means ± SE of one experiment with a total of five mice/group.

The administration of zVAD in PVM-infected mice led to a substantial increase in total lung neutrophil counts in BALF: 9.66 ± 1.78 × 10^4^ cells in zVAD-treated mice as compared to 4.23 ± 1.04 × 10^4^ cells in vehicle-treated mice (*P* < 0.05) (Fig.1A). No statistically significant differences were found in the number of lymphocytes (7.41 ± 2.52 vs. 4.67 ± 1.64 × 10^4^ cells) and macrophages (21.22 ± 3.48 vs. 15.98 ± 4.24 × 10^4^ cells) in the BALF in PVM-infected mice that received zVAD as compared to mice receiving vehicle alone.

We hypothesized that the increase in neutrophil numbers was a result of decreased apoptosis by zVAD. Therefore, we measured apoptosis of recruited neutrophils, using flow cytometry. As compared to vehicle, zVAD treatment significantly decreased the percentage of apoptotic neutrophils in single-cell suspensions from lung tissue (*P* < 0.05) (Fig.1B). In BALF, no significant difference was found in the percentage of apoptotic neutrophils of the BALF of PVM-infected mice receiving zVAD or vehicle (Fig.1B).

### Treatment with zVAD increases the lung cytokine response in PVM-infected mice

**Figure 2:**
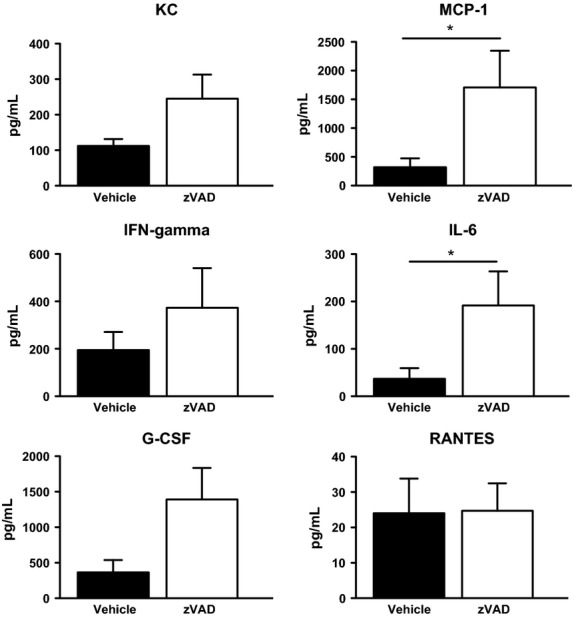
Treatment with zVAD augments the lung cytokine response in PVM-infected mice. Concentrations of KC, MCP-1, IFN-gamma, IL-6, G-CSF, and RANTES in BALF of vehicle or zVAD-treated mice on day 7 after PVM inoculation. *P < 0.05. Data are shown as means ± SE of three independent replicate experiments of three mice/group each, for a total of nine mice/group.

As expected from the increased neutrophil numbers in the lungs, and similar to findings in a Fas-mediated lung injury model (Herrero et al. 2013), the administration of zVAD was associated with enhanced release of several proinflammatory cytokines in the lungs of PVM-infected mice (Fig.2). The PVM-infected mice that were treated with zVAD had statistically significant higher concentrations of MCP-1 and IL-6 in BALF as compared with vehicle-treated mice (*P* < 0.05) (Fig.2). No significant difference was found in the concentrations of KC, IFN-gamma, and G-CSF in BALF in mice that were treated with zVAD as compared to vehicle. The concentrations of IL-12 and IL-13 in BALF were under the detection limit of the assay.

### Treatment with zVAD does not decrease lung caspase-3 activity in PVM-infected mice

**Figure 3:**
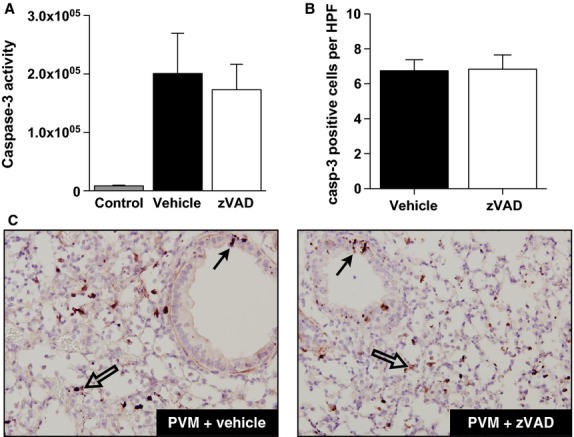
Lung caspase-3 activity in PVM-infected mice is not affected by zVAD treatment. (A) Total lung caspase-3 activity (arbitrary fluorescence units) in vehicle (black bar) or zVAD (white bar)-treated mice on day 7 after PVM inoculation as compared to healthy control mice that received no treatment (gray bar). Data are shown as means ± SE of three independent replicate experiments of 3 mice/group each, for a total of nine mice/group. The data of control mice are based on one experiment with six mice/group. (B) Quantification of cleaved caspase-3-positive epithelial cells (airway and alveolar) per lung tissue section of vehicle (black bar) or zVAD (white bar)-treated mice on day 7 after PVM inoculation. Data are shown as means ± SE of three mice per group. (C) Representative images of cleaved (active) caspase-3 immunohistochemistry in lung tissue of vehicle or zVAD-treated mice on day 7 after PVM inoculation. Closed and open arrows point out cleaved caspase-3-positive airway and alveolar epithelial cells, respectively.

Pneumonia virus of mice infection in mice is associated with a marked increase in total lung caspase-3 activity starting just before and at peak disease severity (Bem et al. 2010c). Both bronchial and alveolar epithelial cells become apoptotic in the late and severe phase of PVM disease (van den Berg et al. 2011). Unexpectedly, zVAD treatment starting at day 5 after inoculation, when early caspase activation is observed, did not affect the overall lung caspase-3 activity on day 7 after PVM infection (Fig.3A). Similarly, no difference in immunohistochemical staining of caspase-3-positive bronchial and alveolar epithelial cells was found between zVAD and vehicle-treated PVM-infected mice (Fig.3B and C).

**Figure 4:**
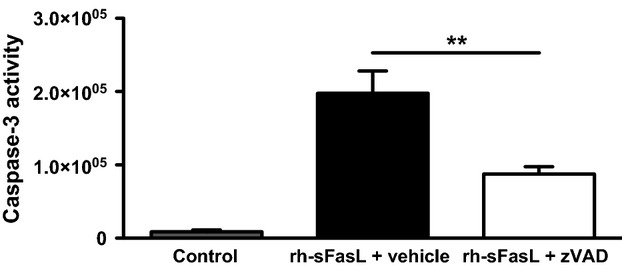
Effect of zVAD on rh-sFasL-induced caspase-3 activity in the lung. Total lung caspase-3 activity (arbitrary fluorescence units) in vehicle (black bar) or zVAD (white bar)-treated mice with rh-sFasL-induced lung caspase-3 activity as compared to control mice that received no treatment (gray bar). **P < 0.01. Data are shown as means ± SE of one experiment with six mice/group.

**Figure 5:**
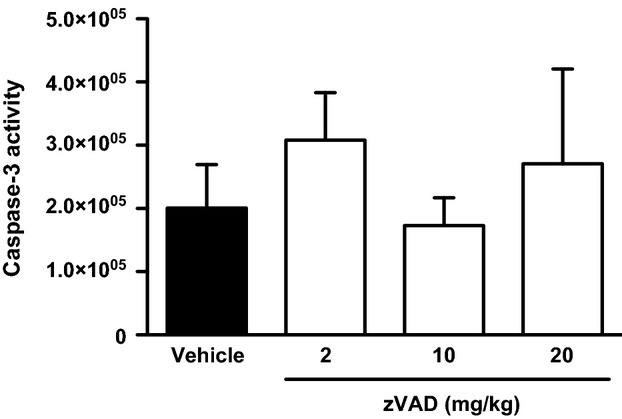
Effect of different doses of zVAD on apoptotic response during PVM disease. Total lung caspase-3 activity (arbitrary fluorescence units) on day 6 in PVM-infected mice (9.6 × 10^3^ copies) that were treated with three different doses of zVAD (2, 10 of 20 mg/kg) or vehicle starting on day 5.5 after infection. Data are shown as means ± SE of 1-3 independent replicate experiments with a total of 3–9 mice/group.

**Figure 6:**
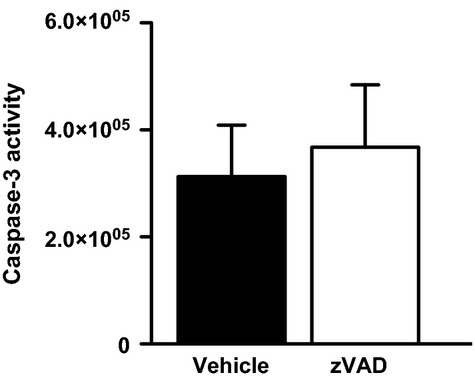
Effect of zVAD on apoptotic response during more severe PVM disease, using a higher inoculum (9.6 × 10^4^ copies) with zVAD treatment on day 4–6. This experiment was performed to confirm the lack of response of zVAD in severe PVM disease. Total lung caspase-3 activity (arbitrary fluorescence units) in vehicle or zVAD-treated mice on day 6 after inoculation with 9.6 × 10^4^ copies of PVM. Data are shown as means ± SE of two independent replicate experiments with a total of 8–9 mice/group.

The dose (10 mg/kg) and route (subcutaneous) of administration of zVAD used in this work were based on recent studies that showed decreased lung epithelial apoptosis and/or lung permeability in two separate models of lung injury (Lipke et al. 2010; Herrero et al. 2013). To exclude potential technical problems with zVAD treatment in our hands, we performed an experiment in which recombinant human soluble Fas ligand (rh-sFasL) was used to induce apoptosis in the lungs. Our zVAD dosing regime was sufficient to induce significant inhibition of caspase-3 activity in rh-sFasL-treated mice (Fig.4). Because of concern that zVAD in the 10 mg/kg dose tested might not be efficient enough to inhibit caspases in the context of a relatively high proapoptotic stimulus as PVM infection, we tested zVAD in a very high dose of 20 mg/kg. Similarly, zVAD in this experiment also failed to modify the lung caspase-3 activity in PVM-infected mice (Fig.5). Furthermore, we studied PVM-infected mice at earlier time-points (3–6 h) after the last zVAD injection and found no differences between the zVAD and vehicle-treated animals (data not shown). Finally, we performed experiments during a more severe course of PVM disease, induced by increasing by one order of magnitude the dose of PVM (9.6 × 10^4^ PVM copies). Again, we found no difference in lung caspase-3 activity in zVAD-treated mice, as compared to mice receiving vehicle alone (Fig.6).

### Treatment with zVAD is not associated with changes in lung viral load, lung permeability, or lung histology

**Figure 7:**
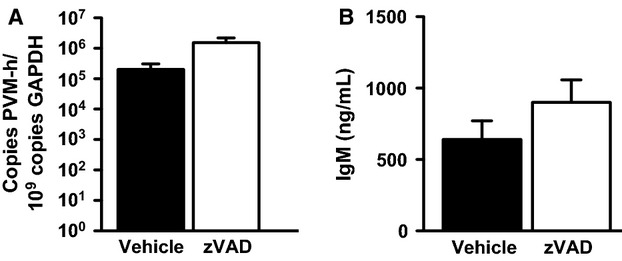
Treatment with zVAD is not associated with changes in viral load or lung permeability in PVM infection. (A) Lung viral load in PVM-infected mice. Total lung viral titers expressed as number of PVM SH copies per 10^9^ GAPDH copies in lung homogenate of vehicle or zVAD-treated mice on day 7 after PVM inoculation. (B) Lung permeability response in PVM-infected mice. Concentrations of IgM in BALF of vehicle or zVAD-treated mice on day 7 after PVM inoculation. Data are shown as means SE of three independent replicate experiments of three mice/group each, for a total of nine mice/group.

**Figure 8:**
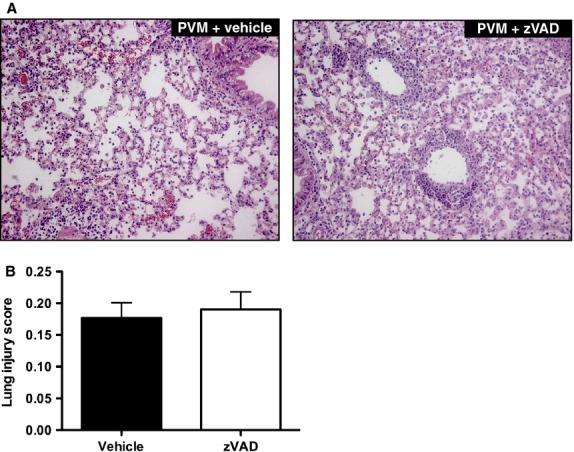
zVAD treatment does not affect lung histopathology during PVM infection. Representative hematoxylin and eosin-stained lung tissue sections (magnification 200×) (A) and lung injury histology scores (B) from vehicle or zVAD-treated mice on day 7 after PVM inoculation. Data are shown as means ± SE of three independent replicate experiments of three mice/group each, for a total of nine mice/group.

In agreement with the observation that zVAD treatment failed to attenuate bronchial or alveolar epithelial cell apoptosis, zVAD treatment had no effect on the lung viral load (Fig.7A), or on lung permeability, as determined by the leakage of the high-molecular weight protein IgM into the alveolar spaces (Cook et al. 1998) (900.6 ± 157.0 ng/mL in zVAD-treated mice versus 640.8 ± 129.7 ng/mL in vehicle-treated mice, *P* = 0.22, Fig.7B). Similar to previous observations (Bem et al. 2010c, 2011), all PVM-infected mice showed marked peri-bronchiolar and alveolar cellular infiltration (Fig.8A). However, no difference was found in the histological lung injury score between the zVAD and vehicle-treated groups (Fig.8B).

### Treatment with zVAD is associated with mild increase in clinical PVM disease

**Figure 9:**
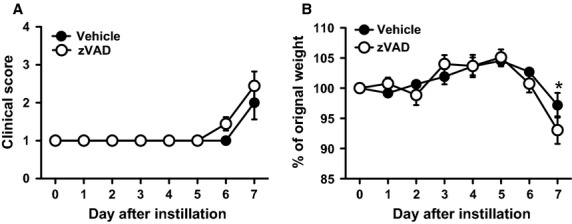
zVAD treatment causes a mild increase in PVM disease severity. (A) Clinical scores of vehicle or zVAD-treated mice assessed daily after PVM inoculation. Data are shown as means ± SE of nine mice/group. (B) Percentage of original body weight of vehicle or zVAD-treated mice assessed daily after PVM inoculation. *P < 0.05. Data are shown as means SE of three independent replicate experiments of three mice/group each, for a total of nine mice/group.

While no statistical significant difference was observed in the clinical scores between PVM-infected mice that were treated with zVAD or vehicle, the weight loss, as a purely objective measure of disease severity, on day 7 after PVM inoculation was increased in zVAD-treated mice as compared to the vehicle-treated mice (Fig.9).

## Discussion

The primary goal of this study was to investigate the effect of the caspase inhibitor zVAD on lung inflammation and injury in mice with severe pneumovirus infection. We found that the administration of zVAD resulted in decreased neutrophil apoptosis in the lungs of PVM-infected mice, and this was associated with increased neutrophil counts and release of proinflammatory cytokines. In contrast, zVAD did not reduce lung epithelial cell apoptosis, and did not affect viral load and lung permeability.

Numerous studies in humans with ARDS and animal ARDS models (Bachofen and Weibel 1982; Matute-Bello et al. 1999; Martin et al. 2005; Bem et al. 2007, 2010b; Herold et al. 2008; Perl et al. 2008) have implicated apoptosis of lung epithelial cells as a major pathogenic event in the development of lung injury. Epithelial cell apoptosis contributes to the loss of the alveolar capillary barrier, which increases lung permeability, resulting in the formation of lung edema. The interesting findings of extensive expression of apoptosis markers in the lung epithelium of children with fatal RSV-LRTD by Welliver and coworkers (Welliver et al. 2007, 2008; Reed et al. 2008), together with similar observations in calves with severe bovine RSV disease and mice with PVM-induced lung injury (Viuff et al. 2002; Bem et al. 2010c), suggest that this type of cell death is also important in severe pneumovirus disease (van den Berg et al. 2013). Importantly, pharmacological interventions aimed at inhibiting proapoptotic signaling pathways in the lungs in animal models of ARDS have been quite successful in protecting against lung histopathological injury, alveolar permeability, and clinical disease (Kawasaki et al. 2000; Matute-Bello et al. 2001, 2005; Le Berre et al. 2004; Perl et al. 2005; Lipke et al. 2010; Herrero et al. 2013; Messer et al. 2013). Our study adds to the body of literature on this topic, however, by showing inefficient and even unfavorable responses to treatment with the caspase inhibitor zVAD in severe pneumovirus-induced lung injury.

In our study, zVAD treatment did not affect lung epithelial caspase-3 activation (classical apoptosis), but instead inhibited apoptosis of recruited neutrophils, leading to increased local neutrophil numbers. The finding of decreased neutrophil apoptosis by zVAD in vivo is in agreement with two animal studies of pleural inflammation (Rossi et al. 2006; D'Avila et al. 2008), and multiple in vitro studies showing that zVAD is able to inhibit apoptosis of neutrophils under various cell culture conditions (Maianski et al. 2002; Luo and Loison 2008; Wardle et al. 2011). However, our observations are in contrast with three studies investigating the effects of zVAD in different animal models of ARDS (Kawasaki et al. 2000; Le Berre et al. 2004; Herrero et al. 2013), including our own control experiment, in which zVAD effectively reduced caspase-3 activity in an independent mouse model of lung injury based on the administration of rh-sFasL. For example, Kawasaki et al. (2000) found decreased apoptosis of structural lung cells, including epithelial cells, in mice with systemic LPS-induced lung injury following exposure to zVAD, and this was associated with better survival. In a rat model of *Pseudomonas aeruginosa* pneumonia, Le Berre et al. (2004) showed reduced apoptosis of lung epithelial cells, and less lung histopathological injury and permeability following treatment with zVAD. Finally, zVAD led to effective inhibition of lung epithelial cell apoptosis, associated with a decrease in lung permeability in Fas-induced lung injury in a recent study by Herrero and coworkers (Herrero et al. 2013). The same group previously showed that zVAD also reduces lung permeability in a mouse model of intratracheal LPS-induced lung injury, presumably by blocking apoptosis of lung epithelial cells (Lipke et al. 2010). Interestingly, in these latter two studies the neutrophil numbers in the lungs were unaffected by zVAD treatment.

From the above studies and our own findings it appears that the antiapoptotic effect of zVAD in the lungs is cell specific and depends on the animal model of injury used. This highlights the complexity of apoptosis-based pharmacological treatments in lung injury (Albertine et al. 2002; van den Berg et al. 2013). Importantly, we used a broad range of doses similar to and higher than the doses used in previous studies (Kawasaki et al. 2000; Le Berre et al. 2004; Lipke et al. 2010; Herrero et al. 2013), and studied the mice at earlier times after zVAD treatment, suggesting that the cause of this differential effect of zVAD in our study was independent of dosing and timing. In addition, we used the same route of zVAD administration as previous studies (Lipke et al. 2010; Herrero et al. 2013), and confirmed the inhibitory effect of zVAD in an independent mouse model of lung injury using rh-sFasL. Likely, the extent, cellular distribution and dynamics of caspase activation in the lungs differ among the various models of lung injury, causing the observed cell-specific effect of zVAD. However, the exact underlying mechanisms of the differential effects of zVAD remain to be elucidated.

In our model of severe pneumovirus disease, the treatment with zVAD led to enhanced lung neutrophil and cytokine inflammation. This is important as it is well recognized that uncontrolled and prolonged lung neutrophil inflammation contributes to ARDS pathophysiology (Ware and Matthay 2000; Martin 2002). Indeed, our findings of moderate exaggeration of weight loss in zVAD-treated PVM-infected mice may be an early reflection of enhanced disease severity as a result of the increased pulmonary inflammation by zVAD. The exaggeration of the release of proinflammatory cytokines, including IL-6, may be the result of prolonged neutrophil lifespan by zVAD in our model. Auto- or paracrine IL-6 signaling of neutrophils may further promote their own survival (Lindemans et al. 2006), thereby creating a spiral toward extensive neutrophilic inflammation. However, in the study of Herrero et al. (2013) zVAD increased the release of pro-inflammatory cytokines, including IL-6 and KC, in Fas-induced lung injury, without causing a change in the lung neutrophil numbers. This suggests that caspase inhibition by zVAD may also promote inflammation uncoupled from its antiapoptotic effect, such as has been found in vitro in Fas stimulated lymphocytes (Scheller et al. 2002).

The most important limitation of our study is that we were unable to investigate the role of lung epithelial cell apoptosis in the disease pathogenesis of severe pneumovirus disease, as zVAD was not effective in inhibiting lung epithelial caspase activity in our model. Therefore, other, more lung epithelial cell-specific, anti-apoptotic treatment strategies need to be exploited in our model in future studies.

In conclusion, zVAD has a primarily proinflammatory effect in PVM-induced lung injury in mice, by enhancing the pulmonary neutrophil and cytokine response, at least in part by inhibiting neutrophil apoptosis. In contrast, zVAD at the doses and times tested is not effective in protecting the lung epithelium from apoptosis in severe PVM disease. We consider zVAD treatment to be potentially harmful in severe pneumovirus disease.
